# Dietary glutamine supplementation prevents mucosal injury and modulates intestinal epithelial restitution following acetic acid induced intestinal injury in rats

**DOI:** 10.1186/1743-7075-10-53

**Published:** 2013-08-07

**Authors:** Forat Swaid, Igor Sukhotnik, Ibrahim Matter, Drora Berkowitz, Christopher Hadjittofi, Yulia Pollak, Alexandra Lavy

**Affiliations:** 1 Department of Surgery, Bnai Zion Medical Center, Haifa, Israel; 2 Department of Gastroenterology, Bnai Zion Medical Center, Haifa, Israel; 3 Laboratory of Intestinal Adaptation and Recovery, The Bruce Rappaport Faculty of Medicine, Technion-Israel Institute of Technology, Haifa, Israel; 4 Elderly Care Department, Queen Elizabeth II Hospital, Welwyn Garden City, United Kingdom; 5 Department Pediatric Surgery, Bnai Zion Medical Center, 47 Golomb St., POB 4940, Haifa 31048, Israel

**Keywords:** Acetic acid, Intestine, Glutamine, Enterocyte proliferation, Enterocyte apoptosis, Crohn’s, Colitis, Inflammation

## Abstract

Beneficial effects of glutamine (GLN) have been described in many gastrointestinal disorders. The aim of the present study was to evaluate the preventative effect of oral GLN supplementation against acetic acid (AA) induced intestinal injury in a rat. Male Sprague–Dawley rats were divided into four experimental groups: control (CONTR) rats underwent laparotomy, control-glutamine (CONTR-GLN) rats were treated with enteral glutamine given in drinking water (2%) 48 hours before and five days following laparotomy, AA rats underwent laparotomy and injection of AA into an isolated jejunal loop, and acetic acid-glutamine (AA-GLN) rats underwent AA-induced injury and were treated with enteral GLN 48 hours before and 5 days following laparotomy. Intestinal mucosal damage (Park’s injury score), mucosal structural changes, enterocyte proliferation and enterocyte apoptosis were determined five days following intestinal injury. Western blotting was used to determine p-ERK and bax protein levels. AA-induced intestinal injury resulted in a significantly increased intestinal injury score with concomitant inhibition of cell turnover (reduced proliferation and enhanced apoptosis). Treatment with dietary GLN supplementation resulted in a decreased intestinal injury score with concomitant stimulation of cell turnover (enhanced proliferation and reduced apoptosis). In conclusion, pre-treatment with oral GLN prevents mucosal injury and improves intestinal recovery following AA-induced intestinal injury in rats.

## Introduction

The identification of factors that prevent mucosal injury during intestinal damage as well as factors that improve intestinal restitution following intestinal injury may reveal new therapeutic strategies for maintaining mucosal integrity of the gastrointestinal (GI) tract, thus improving outcomes in patients with intestinal inflammation. Intracolonic administration of acetic acid (AA) has been previously described as a model of experimental colitis [1]. We recently developed a novel rodent model system to study AA-induced small intestinal injury, whereby acetic acid (0.67 mol/L) was directly administered to a ligated intestinal loop in anesthetized rats, leading to histological injury and alterations in intestinal permeability [2]. AA treatment induced major histopathological changes in the intestinal mucosa, including small, irregular, and distorted villi; epithelial damage; edema of the lamina propria; inflammatory cell accumulation; and hemorrhage. This new model was utilized to study the role of glutamine (GLN) in AA-induced small bowel injury, which is becoming an increasingly relevant topic since this form of GI injury is quite common among patients with Crohn’s disease.

GLN is a non-essential amino acid, produced mainly by muscle, and plays an important role in many physiological and biological processes. Recent evidence suggests that GLN is an important nutrient for rapidly dividing cells such as those found in the immune system and gut [3]. Extensive studies involving various experimental models have established that GLN is an essential respiratory substrate for cells in the small intestinal mucosa, accounting for over one third of the total CO_2_ produced in the small intestine [4]. In addition, GLN exerts a positive effect on gut-associated lymphoid tissue and enhances gut barrier function.

Given the trophic gut effects of GLN, we hypothesized in the present study that this amino acid could prevent intestinal mucosal injury and/or improve intestinal recovery following AA-induced intestinal damage. GLN might stimulate enterocyte turnover via direct stimulation of proliferation or cell migration, or by inhibition of enterocyte apoptosis. Previous studies have shown that intestinal damage results in the appearance of apoptotic and necrotic cells in intestinal epithelium. Furthermore, stimulation of apoptosis is more pronounced in epithelial cells within the upper portions of the villi when compared to the undifferentiated proliferating cells in the crypts of Lieberkühn [5].

The purpose of the present study was to evaluate the effect of dietary GLN supplementation on structural mucosal changes following AA-induced intestinal injury in a rat model and to evaluate the mechanisms by which glutamine influences intestinal recovery, including its effect on enterocyte proliferation and death via apoptosis.

## Materials and methods

### Animals

Male Sprague–Dawley rats weighing 250-350 g were handled in accordance with the guidelines of the Institutional Animal Care and Use Committee (Rappaport Faculty of Medicine, Technion, Haifa, Israel). Rats were housed individually in stainless steel cages and were acclimatized to standard conditions of room temperature (25°C) with alternating 12-hour light–dark cycles and fed standard rat chow and water *ad libitum* during a minimum stabilization period of five days.

### Experimental design

Rats were randomly assigned to one of four experimental groups: Control (CONTR) rats underwent laparotomy, isolation of jejunal loop and intraluminal injection of normal saline. Control-glutamine (CONTR-GLN) rats were treated with enteral GLN given in drinking water (2%) 48 hours before and 5 days following laparotomy. Control-acetic acid (C-AA) rats underwent laparotomy, isolation of jejunal loop and intraluminal injection of 2 ml (0.67 mol/L) AA as previously described [2]. Finally, acetic acid-glutamine (AA-GLN) rats underwent AA-induced injury (similarly to C-AA rats) and were treated with enteral glutamine 48 hours before and 5 days following operation (similarly to C-GLN rats).

### Surgical procedure

The rats were preoperatively fasted for 12 hours. Operative procedures were performed using standard sterile technique under general anesthesia with ketamine (intraperitoneally, 90 mg/kg) and xylazine (intraperitoneally, 10 mg/kg). The abdomen was accessed through a midline incision. C rats underwent laparotomy, isolation of jejunal loop and intraluminal injection of normal saline. In AA rats, after laparotomy and isolation of jejunal loop, atraumatic vascular clamps were used to occlude the isolated intestinal loop, and 2 ml of AA (0.67 mol/L) was injected into the lumen for 10 minutes. During the period of injury the abdominal wall incision was kept approximated to prevent fluid and heat loss. After a 10-minute period of damage, AA was evacuated and the intestinal occlusions were released. The intestines were carefully replaced in the abdomen, and the incision was covered with moist gauze. The rats were placed on a heating blanket for the duration of the procedure. They were subsequently resuscitated with a 3 ml intraperitoneal injection of warm 0.9% saline, and the incision was closed with a Dexon S Polyglycolic Acid 3–0 (TYCO Healthcare, Mansfield, MA) running suture. The rats were then allowed to awake with free access to water and food.

In the pilot study, changes on days one, three, and five were investigated after AA injection. Since the histopathological intestinal changes after one and three days represented mainly acute injury, findings at five days were regarded as representative of the chronic damage which resembles inflammatory bowel disease in humans more accurately. Time of sacrifice was therefore established at five days after intestinal damage. The rats were re-anesthetized with intraperitoneal pentobarbital (75 mg/kg) and were sacrificed by open pneumothorax. Two intestinal segments (proximal jejunum and distal ileum; 10 cm each) were removed and flushed with cold saline before recording wet weight. The mucosa was scraped from the underlying tissue with a glass slide and weighed. Bowel and mucosal weights were calculated as mg/cm-bowel-length/100 g-body-weight.

### Histological examination

Histological sections were prepared from the proximal jejunum and distal ileum. Segments of small bowel were fixed for 24 hours in 4% buffered formalin, cleared in xylene, and processed into standard paraffin blocks. Five-micron tissue slices were deparaffinized and were stained with H&E. The degree of intestinal tissue injury was evaluated on a grading scale from 0 to 8 as described previously by Park et al. [6]: 0 - normal mucosa, 1 - subepithelial space at villus tip, 2 - more extended subepithelial space, 3 - epithelial lifting along villus sides, 4 - denuded villi, 5 - loss of villus tissue, 6 - crypt layer infarction, 7 - transmucosal infarction, 8 - transmural infarction.

Villus height and crypt depth were measured in ten villi and crypts, using the Image-Pro Plus 4 image analysis software (Media Cybernetics, Baltimore, Maryland, USA).

### Enterocyte proliferation

To determine enterocyte proliferation, rats were injected with standard 5-bromodeoxyuridine (5-BrdU) labeling reagent (Zymed Laboratories Inc. CA) at a dose of 1 ml per 100 g body weight 90 minutes before sacrifice. Five-micrometer paraffin-embedded slices (5 μm) were deparaffinized with xylene, rehydrated with graded alcohol, and stained with a biotinylated monoclonal anti-BrdU antibody system using the BrdU Staining Kit (Zymed Laboratories Inc. CA). The proliferation index was defined as the ratio of crypt cells staining positively for BrdU per ten crypts.

### Enterocyte apoptosis

Apoptotic cells were identified using immunohistochemical analysis for caspase-3. Caspase-3 (CPP32/YAMA) is a marker for the early apoptotic phase. Five-micrometer paraffin-embedded sections were dewaxed and rehydrated with xylene and graded alcohol. Tissue sections were microwave-pretreated in 10 mM citrate buffer (pH 6.0). Immunohistochemical analysis for caspase-3 was performed using the streptavidin-biotin-peroxidase method according to manufacturer protocols. After the blocking of endogenous biotin activity, tissue sections were incubated with primary antibodies (Caspase-3 cleaved concentrated polyclonal antibody; dilution 1:100; Biocare Medical, Walnut Creek, CA) for 1 hour at room temperature, followed by incubation with secondary antibodies (human-absorbed, biotinylated, affinity-purified antibody) for 20 minutes. The DAB was used to create an intense brown deposit around the antigen-antibody-enzyme complex in the sample. For each group, the number of stained cells was counted in two bowel cross sections. The apoptotic index (AI) was defined as the number of apoptotic cells per ten villi. Control kit from Eurogentec, EGT Group.

#### Western blotting

Tissue was homogenized in RIPA lysis buffer containing 50 mM Tris–HCl (pH 7.4), 150 mM NaCl, 1% NP-40, 2 mM EDTA, supplemented with a cocktail of protease and phosphatase inhibitors. Protein concentrations were determined by Bradford reagent according to the manufacturer’s instructions. Samples containing equal amounts of total protein (30 μg) were resolved by SDS-PAGE under reducing conditions. After electrophoresis, proteins were transferred to a PVDF membrane and probed with various primary antibodies to anti-bax antibody (1:200 dilution, sc-493), anti-phospho-ERK antibody (1:2500 dilution, sc-7383), and anti-β-Tubulin (1:5000 dilution, Sigma T6557) was used for the protein normalization. Horseradish peroxidase-conjugated secondary antibody was purchased from Jackson ImmunoResearch Laboratories Inc. (West Grove, PA) and an enhanced chemiluminescent substrate from Biological Industries (Kibbutz Beth HaEmek, Israel). The optical density of the specific protein bands was quantified by using a densitometer (Vilber Lourmat, Lion, France).

### Statistical analysis

The data are expressed as the mean ± SEM. A paired Student’s *t*-test and the non-parametric Kruskal-Wallis ANOVA test were used as indicated. P < 0.05 was considered statistically significant.

## Results

### Body weight

**Figure 1:**
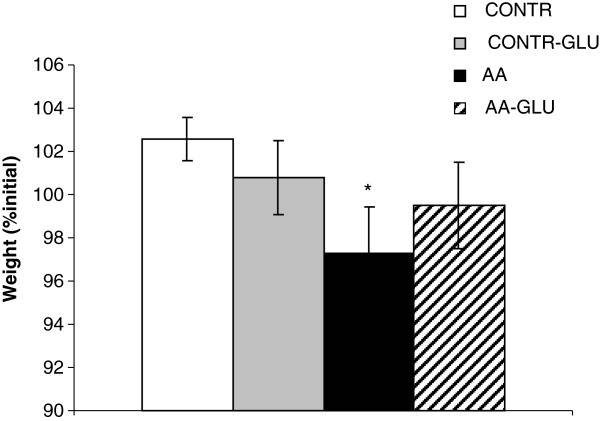
**Effect of acetic acid intestinal injury and glutamine supplementation on body weight changes.** Values are mean ± SEM. CONTR-control; AA- acetic acid; GLN- glutamine. * P < 0.05 vs CONTR rats, † P < 0.05 AA-GLN vs AA rats.

Treatment of sham rats with enteral GLN (CONTR-GLN group) did not significantly change final body weight compared to CONTR rats (Figure 1). AA rats demonstrated a significant decrease in final body weight (97 ± 2 vs. 103 ± 1% initial, p < 0.05) compared to CONTR rats. Enteral GLN supplementation (AA-GLN group) resulted in a trend toward increase in final body weight compared to AA animals; however, this trend did not achieve statistical significance.

### Intestinal injury score

**Figure 2:**
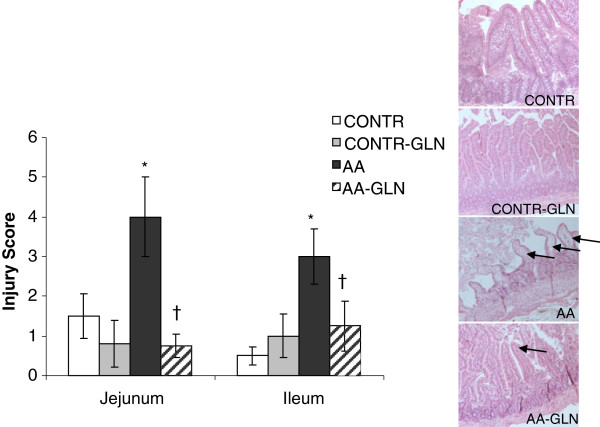
**Effect of acetic acid and glutamine on the microscopic intestinal appearance.** As expected, control rats demonstrate a normal histologic architecture. AA rats show extended subepithelial space and epithelial lifting along villus sides (Park score 3–4). AA-GLN rats exhibited a less marked subepithelial space at villus tip (Park score 1–2). Values are mean ± SEM. CONTR-control; AA- acetic acid; GLN- glutamine. * P < 0.05 vs CONTR rats, † P < 0.05 AA-GLN vs AA rats.

Whereas the jejunum and the ileum of CONTR and CONTR-GLN rats exhibited normal mucosal architecture with intact villi, AA animals exhibited a significant histologic injury in damaged jejunum and less significant changes in ileum (Figure 2) as well as an inflammatory cells accumulation, edema and hemorrhage that seen mainly at site of the injury. AA rats (Group C) demonstrated a significant increase in the mean intestinal injury grade in jejunum (three-fold, p < 0.05) and ileum (six-fold, p < 0.05) compared to CONTR rats. Pre-treatment with oral GLN prevented intestinal mucosal injury caused by AA. AA-GLN rats attracted significantly lower histological scores in jejunum (four-fold decrease, p < 0.05) and ileum (three-fold, p < 0.05) compared to AA rats. However, AA-GLN rats demonstrated a less effects on inflammatory cells accumulation, edema and hemorrhage at site of the injury.

### Intestinal mucosal parameters

**Figure 3:**
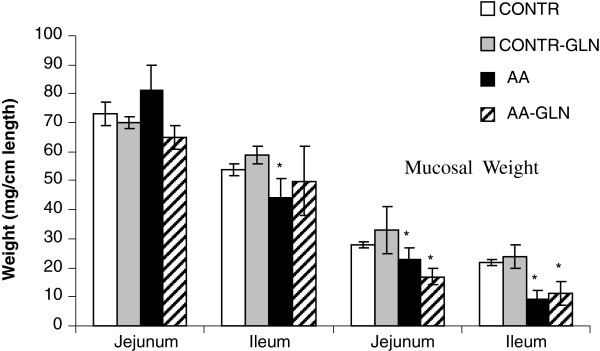
**Effect of enteral glutamine on macroscopic intestinal appearance during acetic acid-induced intestinal damage.** Values are mean ± SEM. CONTR-control; AA- acetic acid; GLN- glutamine. * P < 0.05 vs CONTR rats, † P < 0.05 AA-GLN vs AA rats.

Treatment of control animals with enteral glutamine (CONTR-GLN group) did not significantly change bowel and mucosal weight compared to control animals (C group). 5 days after intestinal damage, there was a decrease in intestinal wall thickness and gut diameter. AA rats had a significantly lower bowel weight in ileum (44 ± 7 vs. 54 ± 2, p < 0.05) and mucosal weight in both jejunum (23 ± 4 vs. 28 ± 1, p < 0.05) and ileum (9 ± 3 vs. 22 ± 1, p < 0.05) compared to control animals (C group) (Figure 3). AA-GLN rats demonstrated a trend toward increase in ileal bowel and mucosal weight; however, this trend did not achieve statistical significance.

### Microscopic mucosal parameters

**Figure 4:**
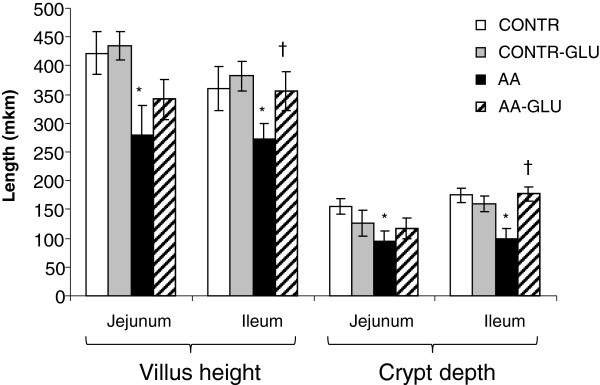
**Effect of enteral glutamine on microscopic intestinal appearance during acetic acid-induced intestinal damage.** Values are mean ± SEM. CONTR-control; AA- acetic acid; GLN- glutamine. * P < 0.05 vs CONTR rats, † P < 0.05 AA-GLN vs AA rats.

AA-rats demonstrated a significant decrease in jejunal (279 ± 52 vs. 421 ± 37 μm, p < 0.05) and ileal (272 ± 28 vs. 361 ± 39 μm, p < 0.05, p < 0.05) villus height as well as in jejunal (95 ± 17 vs. 155 ± 14 μm, p < 0.05, p < 0.05) and ileal (98 ± 20 vs. 175 ± 22 μm, p < 0.05, p < 0.05) crypt depth compared to CONTR rats (Figure 4). Oral glutamine supplementation (AA-GLN group) caused a significant increase in ileal villus height (356 ± 28 vs. 272 ± 28 μm, p < 0.05 p < 0.05) and crypt depth (177 ± 12 vs. 98 ± 20 μm, p < 0.05) as well as a trend toward increase in jejunal villus height and crypt depth; however, this trend was not statistically significant.

### Enterocyte proliferation and apoptosis

**Figure 5:**
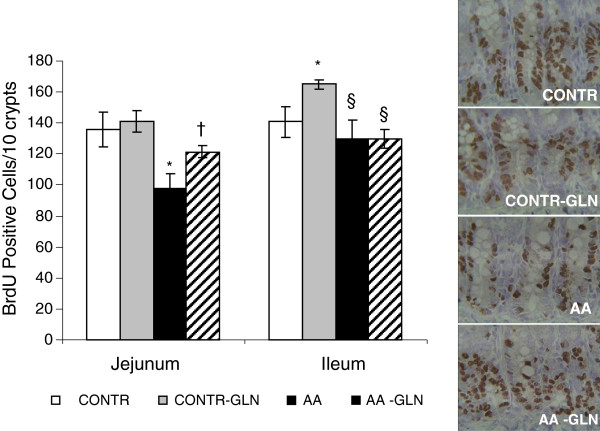
**Effect of acetic acid and oral glutamine on crypt cell proliferation.** 5-BrdU incorporation into proliferating jejunal and ileal crypt cells was detected with a goat anti-BrdU antibody. The representative sections (on the right side) demonstrate that cell proliferation decreased following AA-damage compared to control animals. Following administration of oral glutamine, AA-rats demonstrated a marked increase in a number of proliferating cells compared to AA non-treated animals. Values are mean ± SEM. CONTR-control; AA- acetic acid; GLN- glutamine. * P < 0.05 vs CONTR rats, † P < 0.05 AA-GLN vs AA rats.

Treatment of control rats with glutamine (CONTR-GLN group) led to a mild increase in cell proliferation rates in ileum (165 ± 3 vs. 141 ± 10 BrdU positive cells/10 crypts, p < 0.05) compared to CONTR rats as well as in a trend toward increase in cell proliferation rates in jejunum; however, this trend was not statistically significant (Figure 5). AA-induced intestinal damage (C-AA rats) resulted in a significant decrease in the enterocyte proliferation index in jejunum (98 ± 10 vs. 136 ± 11 BrdU positive cells/10 crypts, p < 0.05) and ileum (130 ± 12 vs. 141 ± 10 BrdU positive cells/10 crypts, p < 0.05) compared to control animals. Oral glutamine administration (AA-GLN group) induced a significant increase in the proliferation index in ileum (122 ± 4 vs. 98 ± 10 BrdU positive cells/10 crypts, p < 0.05) compared to AA rats. Both AA and AA-GLN rats demonstrated a lower cell proliferation rates compared to CONTR-GLN rats.

**Figure 6:**
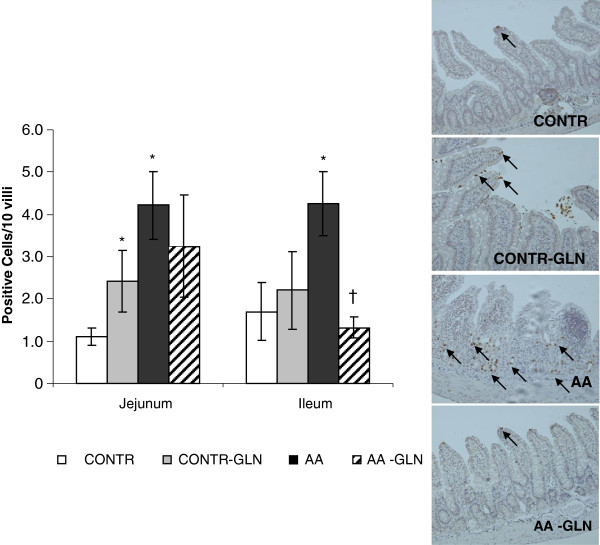
**Effect of acetic acid and oral glutamine on enterocyte apoptosis.** Immunohistochemistry for caspase-3 was used to determine enterocyte apoptosis. The representative sections (on the right side) demonstrate that the number of the Caspase-3 positive cells increased significantly following AA compared to control animals and decreased significantly after treatment with glutamine. Values are mean ± SEM. CONTR-control; AA- acetic acid; GLN- glutamine. * P < 0.05 vs CONTR rats, † P < 0.05 AA-GLN vs AA rats.

CONTR-GLN rats showed a significant increase in cell apoptosis in jejunum (2.4 ± 0.7 vs. 1.1 ± 0.2 Caspase-3 positive cells/10 villi, p < 0.05) compared to CONTR rats (Figure 6). AA-induced intestinal damage (Group C) resulted in a significantly greater number of Caspase-3 positive cells appearing in the villi of jejunum (4.2 ± 0.3 vs 2.8 ± 0.6 apoptotic cells/10 villi, p < 0.05) and ileum (4.3 ± 0.84 vs. 1.7 ± 0.6 apoptotic cells/10 villi, p < 0.05) compared to control animals (Figure 3). Pre-treatment with oral glutamine (AA-GLN) resulted in a significant decrease in apoptotic index in ileum (three-fold decrease, p < 0.05) compared to AA rats, as well as in a trend toward decrease in cell apoptosis rates in jejunum; however, this trend was not statistically significant.

#### Western blotting

**Figure 7:**
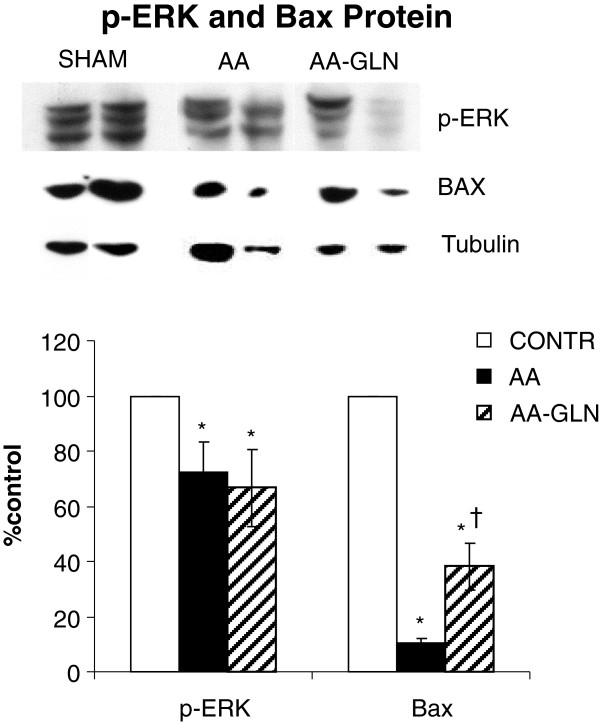
**Effect of acetic acid and oral glutamine on expression of p-ERK, and bax protein (Western blot) in intestinal mucosal samples.** Values are mean ± SEM. CONTR-control; AA- acetic acid; GLN- glutamine. * P < 0.05 vs CONTR rats, † P < 0.05 AA-GLN vs AA rats.

Decreased cell proliferation rates in AA animals (Groups B and C) were accompanied by decreased levels of p-ERK protein. Interestingly, an increased cell apoptosis was accompanied by decreased bax-protein levels in AA animals (Groups B and C) compared to control animals. Treatment with glutamine (Group C) did not change significantly the levels of p-ERK and Bax protein compared to AA-nontreated animals (Group B) (Figure 7).

## Discussion

The inflammatory bowel diseases, including Crohn’s disease (CD) and ulcerative colitis (UC), are chronic inflammatory disorders characterized by inappropriate and persistent activation of the intestinal mucosal immune system [7]. Patients with CD suffer the consequences of a transmural inflammatory process (classically apparent as “skip lesions”) and are thus at risk of developing fistulae, whereas colonic inflammation in UC is continuous, extending from the rectum proximally. Different animal models of colitis and colitis-associated cancer have proven in selected circumstances to be relevant to the pathogenesis of these disorders in humans. Two of the most widely used non-genetic colitis models are the dextran sodium sulfate (DSS)-induced chemical injury model, and the trinitrobenzene sulfonic acid (TNBS) hapten-induced model [8-10]. DSS in drinking water induces an acute colitis within 5 days of exposure, and can also be utilized to mimic chronic colitis following repeated exposure [10,11]. TNBS is administered by enema and results in a hapten-induced, interleukin-12 (IL-12) driven colitis [12]. As summarized in Rosenstiel et al. [13], some mouse models correlate well with human disease risk loci or associated pathways, whereas others do not yet have clear relevance to human clinical syndromes.

We recently developed a novel rodent model system to study AA-induced small intestinal injury, by administering AA (0.67 mol/L) directly to a ligated intestinal loop of anesthetized rats that may be relevant to the pathogenesis of small intestinal chronic inflammation like Crohn’s disease in humans. In the pilot study one, three and five-day changes were investigated after AA injection. After one and three days, the histopathological changes in the intestine represented mainly acute injury, whereas after five days we observed the signs of chronic damage that resemble human inflammatory bowel disease more accurately. Therefore, five days after intestinal damage was chosen as the time of sacrifice. In the current study, the effects of dietary GLN supplementation on structural mucosal changes following AA-induced intestinal injury were evaluated. There is a growing body of evidence to suggest that various nutrients may improve mucosal recovery after gut damage. GLN is a non-essential amino acid, which is produced mainly by the muscle, and which plays an important role in many physiological and biological processes. GLN is the primary metabolic fuel of the small amino acid pool in the body, and has been shown to be an essential metabolic component of the proliferative response of enterocytes. While in the large bowel the short chain fatty acid butyrate is the preferred fuel, several experimental studies have indicated that GLN is one of the preferred fuel sources for the small intestine [14]. Extensive studies in various experimental models have established the positive effect of GLN on the small intestinal mucosa following intestinal damage [15-20]. However, studies on the mechanisms by which this amino acid improves intestinal recovery following intestinal injury are both few and controversial. GLN might stimulate mucosal recovery via direct stimulation of proliferation or cell migration, or via inhibition of enterocyte apoptosis. Alternatively, it might exert its pro-adaptive effect by stimulating the release of various trophic agents or by altering absorption and secretion of nutrients. In a recent review, Mok et al. provide a critical appraisal of the literature on gutamine supplementation in various conditions or illnesses that affect children, from neonates to adolescents, including inflammatory bowel disease [21]. They state that whereas animal models of IBD have indicated potentially beneficial effects of supplemental GLN, clinical studies of GLN in inflammatory bowel disease have been less encouraging. The initial trial found no evidence that GLN enriched polymeric diet is of any benefit over standard polymeric diet in the treatment of children with active Crohn’s disease as well as there were no differences in remission rates, changes in platelet count, orosomucoid level, or weight.

In the present study, we evaluated the consequences of oral GLN in preventing mucosal injury by AA and on gut recovery following injury. Alterations in bowel and mucosal weights, as well as histological appearance were evaluated. Intestinal mucosal parameters were calculated per cm of bowel weight. BrdU was used in our experiment to determine an index of crypt cell proliferation. This analogue of thymidine is incorporated into the DNA of proliferating cells during the S-phase of the cell cycle. Immunohistochemistry for caspase-3 was used to characterize enterocyte apoptosis. Our data demonstrated that injection of AA produced severe small intestinal damage, an effect which was most prominent in the isolated proximal segment but which was almost absent in the distal ileum. This is evident from the increased Park intestinal injury score, observed in both jejunum and ileum; however, the damaging effect was more significant in the jejunum. The intestinal injury in this group was intermediate (Grade 4 in jejunum and grade 2 or 3 in ileum), with no single animal showing very severe tissue damage. Additionally, intestinal damage led to intestinal mucosal hypoplasia, apparent as decreased bowel and mucosal weight. A trend toward increase in bowel weight in jejunum in AA rats with histological evidence of enhanced vascular permeability suggests underlying edema with vascular engorgement rather than mucosal hyperplasia. Our data show that enterocyte proliferation was down-regulated while cell death via apoptosis increased significantly in both jejunum and ileum during intestinal damage. A decreased rates of cell proliferation were accompanied by increased p-ERK protein levels. The extracellular signal-related kinase (ERK) is one of the MAPK signaling pathways triggered by cytokines or growth factors and regulates various cellular activities, such as gene expression, mitosis, cell proliferation, differentiation and apoptosis. A more significant increase in cell death was observed in the proximal jejunum (area of damage). Less significant damage to the distal ileum may be due to a lower achievable concentration of AA in distal segments. Our data also demonstrated that programmed cell death is one of the pathways leading to mucosal damage, in consistence with data from previous studies [22-24]. Interestingly, the expression of pro-apoptotic Bax protein was down-regulated in AA rats compared to sham animals despite the increased rates of cell apoptosis. We believe that the decreased Bax expression creates a higher resistance of enterocytes to apoptosis. The other explanation of this phenomenon may be the different rates of apoptosis at multiple time points following intestinal damage. Consistent with this concept, is the fact that in the first 2–3 days after injury the stimuli for adaptation may suppress programmed cell death in an attempt to increase the cell mass. At this time point down-regulation of the Bax protein may drive decreased cell apoptosis. Five days after bowel damage, when the bulk of immature enterocyte appears, cell apoptosis increases significantly in order to reach a new homeostatic point during intestinal adaptation, in order to promote disposal of genetically aberrant stem cells and to prevent tumorogenesis. At this time point, down-regulation of Bax mRNA coincides with decreased number of Caspase-3 positive cells. These findings suggest an important role for the differential regulation of apoptosis related genes as coordinators of the early increase in cell apoptosis after intestinal damage. Both reduced cell proliferation and enhanced cell apoptosis in the current study indicate reduced enterocyte turnover, which could explain mucosal hypoplasia after AA-induced damage.

In this study, GLN treatment had a beneficial effect in preventing intestinal damage. Administration of oral GLN ameliorated AA-associated tissue damage. Intestine from GLN-treated rats exhibited reversal of villus epithelium loss and injury, as well as a decrease in Park injury score. In addition, AA-induced mucosal hypoplasia was partly ameliorated by oral GLN administration. This was evident as increased bowel and mucosal weight, which is characteristic of tissues undergoing increased cell proliferation or repair. Histologically, villus height and crypt depth increased in response to GLN administration, suggesting increased absorptive surface area. Preserved cell viability following treatment with glutamine leads to enhanced enterocyte turnover and improves gut recovery. The strong increase in the proliferation of crypt cells in the intestinal mucosa of glutamine treated rats seen here is of much significance and correlates with the crypt depth observed. The number of caspase-3-positive cells decreased significantly following GLN administration, suggesting that GLN exerts its positive effect on intestinal recovery through both stimulation of proliferation and inhibition of apoptosis. The positive effect of GLN on non-stressed intestinal mucosa including its stimulating effect on enterocyte proliferation has been extensively described in various experimental studies; however, the molecular mechanisms whereby it enhances intestinal cell growth have not been clearly elucidated. GLN was found to prevent 5-fluorouracil-induced apoptosis and necrosis in an undifferentiated crypt intestinal cell lines [25]. Our findings demonstrate that treatment with glutamine appears to be considerably less effective in activating the MAPK pathway. Further experiment are required to determine whether other pathways (Like PI3K/Akt signaling) may be responsible in transducing increased cell proliferating activity following GLN administration. A recent study has demonstrated that activation protein kinase D (PKD) and phosphatidylinositol 3-kinase (PI3K)/Akt is an important contributor to GLN-mediated intestinal cell survival [26].

Finally, the present study demonstrates that oral GLN attenuates intestinal mucosal injury and accelerates intestinal recovery following AA-induced intestinal damage in rats. This benefit correlates with an increase in enterocyte proliferation and decreased cell apoptosis in intestinal mucosa.

## Competing interests

The authors declare that they have no competing interests.

## Authors’ contributions

The authors declare that they have no conflict of interests. The work was supported in part by the Technion Research Fund (No 1010587). FS, IS and YP carried out the animal study, performed immunohistochemistry, and were responsible for data collection. IM, DB, IS and AL participated in the sequence alignment, carried out the statistical analysis and drafted the manuscript. CH drafted the manuscript. All authors read and approved the final manuscript.
